# Rational and design of the FREESCA study: A multicenter prospective study assessing the prognostic value of continuous glucose monitoring during hospitalization for acute coronary syndrome

**DOI:** 10.1016/j.ahjo.2026.100950

**Published:** 2026-10-19

**Authors:** Sarah Tournayre, Edouard Gerbaud, Pierre Poustis, Bogdan Catargi, Christel Voinot, Meyer Elbaz, Pierre Gourdy, Benoit Lattuca, Véronique Taillard, Patrick Henry, Jean-François Gauthier, Frédéric Georger, Jean-Michel Andrieu, Etienne Puymirat, Fabien Huet, Gwenaelle Arnault, Nicolas Nagot, François Roubille, Ariane Sultan

**Affiliations:** a Nutrition-Diabetes Department, Montpellier University Hospital, Centre Hospitalier Universitaire de Montpellier, 191 Avenue Du Doyen Gaston Giraud, Montpellier, 34295, France; b Cardiology Intensive Care Unit and Interventional Cardiology, Hôpital Cardiologique du Haut-Lévêque, Pessac, France; c Centre Hospitalier de Pau, Pau, France; d Department of Cardiology, Toulouse University Hospital, France; e CHU de Toulouse & UMR1297/I2MC, Université de Toulouse, Toulouse, France; f Cardiology Department, Nimes University Hospital, Montpellier University, Nimes, France; g Department of Cardiology, University Hospital of Lariboisiere, Université Paris-Cité, (Assistance Publique des Hôpitaux de Paris, AP-HP), Paris, 75010, France; h Cardiology Unit, Beziers Hospital, Béziers, France; i Endocrinologie- diabetes departement, Beziers hospital, 1 rue valentin Huy, Beziers, 34500, France; j Assistance Publique-Hôpitaux de Paris (AP-HP), Hôpital Européen Georges Pompidou, Department of Cardiology, Paris, 75015, France; k Université Paris-Cité, Paris, 75006, France; l Intensive Cardiac Care Unit, Cardiology Department, CH Bretagne Atlantique, Vannes, France; m CH Bretagne Atlantique, Vannes, France; n Inserm U1046, CNRS UMR, 9214, INI-CRT, PhyMedExp, Cardiology Department, University of Montpellier, France; o Department of Medical Information, Epidemiology and Health Data, Montpellier University Hospital, University of Montpellier, France

## Abstract

**Background:**

Hyperglycemia and glycemic variability are recognized as major prognostic factors after acute coronary syndrome (ACS), yet most studies rely on single-point glucose measurements or short-term monitoring.

**Aim:**

To describe the rational and the design of the FREESCA study, which aims to evaluate the prognostic value of glycemic parameters recorded by a professional continuous glucose monitoring (CGM) device during the index hospitalization for ACS.

**Methods:**

FREESCA is a prospective, observational, multicenter study planned to enroll 850 adults admitted to intensive cardiac care units for ACS (STEMI or NSTEMI). A Freestyle Libre Pro sensor® is placed at admission and records interstitial glucose continuously throughout hospitalization. Parameters include mean glucose, time in range (TIR), time above range (TAR), time below range (TBR), coefficient of variation (CV), mean amplitude of glycemic excursions (MAGE), and glucose management indicator (GMI). The primary endpoint is the occurrence of major adverse cardiovascular events (MACE) at 12 months.

**Conclusions:**

FREESCA is one of the largest studies to integrate full-hospitalization CGM data in ACS patients regardless of diabetes status, aiming to develop a robust prognostic model.

**Trial registration:**

ClinicalTrials.gov identifier [NCT06014112].

Rational and design of the FREESCA study.

**Figure:**
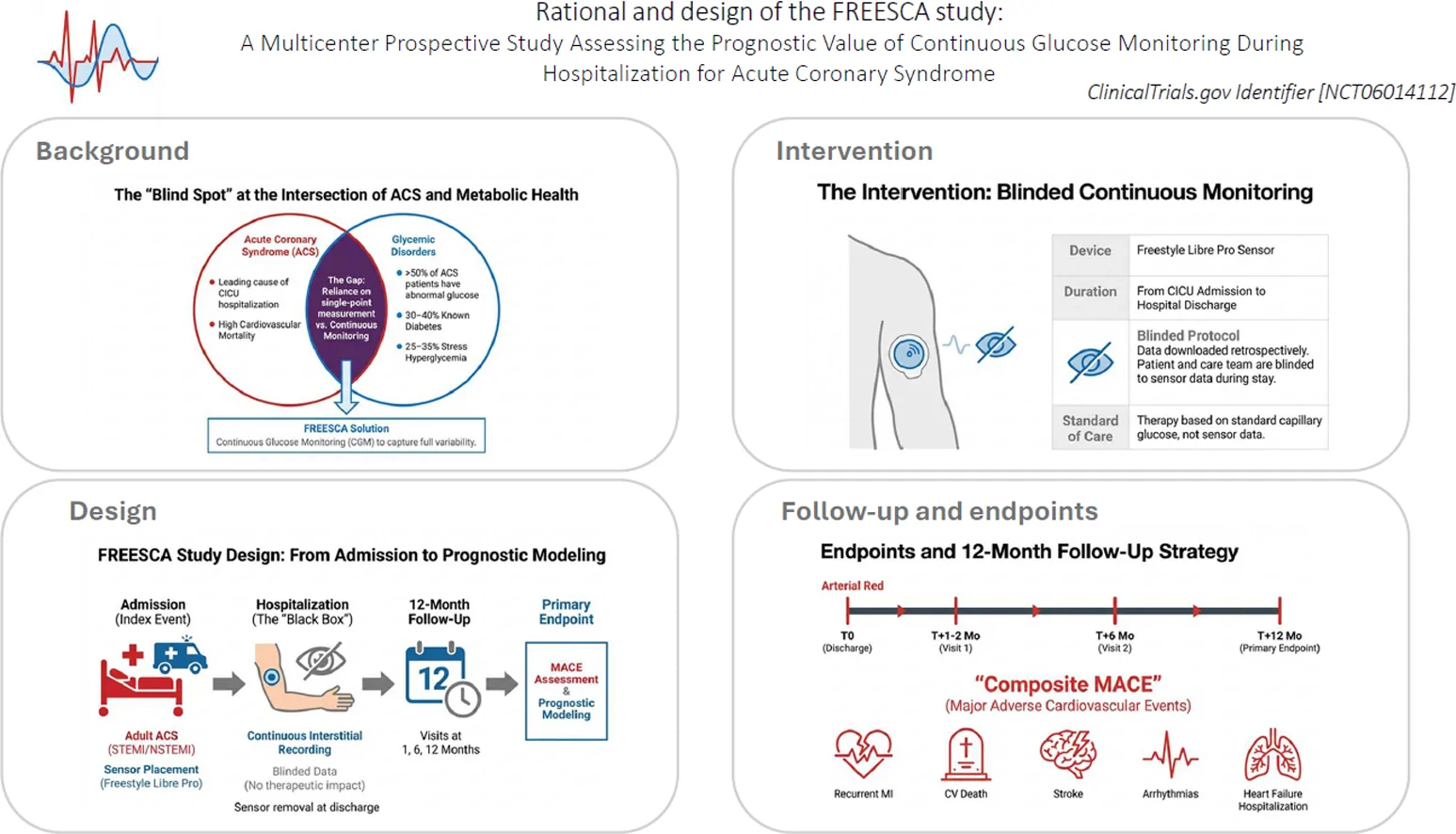

## 1 Background

Cardiovascular mortality remains the leading cause of death in the world with cancer, depending on the country. The leading cause of cardiovascular mortality remains ischemic pathology, mainly related to acute coronary syndromes (ACS). ACS is by far the leading cause of hospitalization in cardiac intensive care units (CICUs) [1]. The average length of stay for a patient with ACS is 4 to 7 days in the French FAST-MI [2]. Beyond the cardiovascular pathology, it is well described that disorders of glycemic regulation are very frequent in patients hospitalized for ACS. Thus, in more than 50% of cases, abnormal blood glucose levels are observed, with diabetes in 30 to 40% of cases, and fasting hyperglycemia or impaired glucose tolerance in 25 to 35% of cases [3] with specific problems to manage, as recently underlined in an expert consensus [4].

From a metabolic point of view, several situations can be distinguished:

– Hyperglycemia occurs in non-known diabetic ACS subjects:

It may be indicative of type 2 diabetes mellitus (T2DM) (fasting blood glucose≥1.26 g/L with HbA1c ≥ 6.5%) or stress hyperglycemia. Indeed, certain stressful situations, including ACS, can induce disturbances in glycemic regulation, as the so-called “stress hyperglycemia”. The threshold values chosen to define stress hyperglycemia vary according to the learned societies. The ADA (American Diabetes Association) defines stress hyperglycemia as a blood glucose level above 1.40 g/L (7.8 mmol/L) [5], [6]. The French Diabetes and Cardiology Societies define stress hyperglycemia as a blood glucose level above 1.80 g/L (10 mmol/L) on admission [7] and the National Institute for Health and Care Excellence (NICE) for blood glucose levels above 2 g/L (11 mmol/L) [8]. Its prevalence is therefore variable, from 9 to 16% depending on the threshold values chosen.

– Hyperglycemia occurs in known diabetic ACS subjects:

According to the joint SFD (French-speaking diabetes society)/SFC (French society of cardiology) recommendations, hyperglycemia is defined as a blood glucose level of more than 1.8 g/L in a known diabetic patient and warrants appropriate management according to the level of hyperglycemia.

Numerous studies have investigated the relationship between blood glucose levels and prognosis for acute coronary syndrome. However, the inclusion criteria are heterogeneous between studies (ACS ST− or ST+, people living with or without diabetes, dialysis patients, patients with hypoglycemia or hyperglycemia…) [9], [10].

FREESCA aims to fill these gaps by evaluating continuous glycemic profiles (Fig. 1) during the entire hospitalization for ACS and their association with cardiovascular outcomes at one year.

**Fig. 1:**
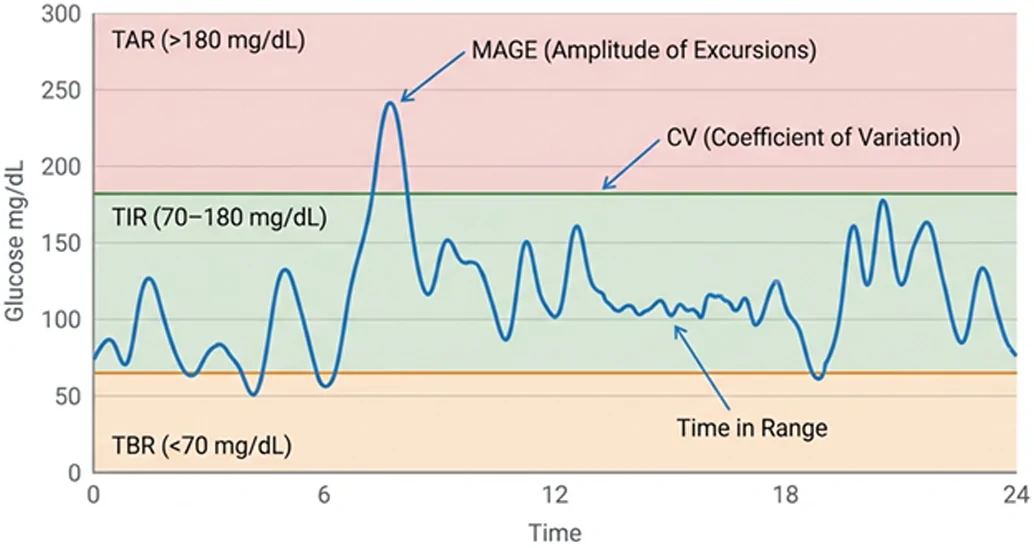
Beyond HbA1c: capturing glycemic variability.

## 2 Material and methods

### 2.1 Study design

FREESCA is a prospective, observational, multicenter cohort study conducted in 10 French cardiology centers. Consecutive patients hospitalized for acute coronary syndrome are enrolled during the index hospitalization.

The first patient was included on 6 November 2023, and the planned inclusion period extends until 6 May 2027, corresponding to a total enrollment duration of 42 months.

Continuous glucose monitoring is performed using FreeStyle® devices, which are provided free of charge by Abbott®, with no involvement of the manufacturer in the study design, data collection, analysis, or interpretation of the results.

Clinical, biological, and CGM-derived data are prospectively collected using a secure electronic case report form (eCRF) developed with Ennov Clinical® software.

The study is registered on ClinicalTrials.gov under the identifier NCT06014112.

### 2.2 Study timeline

See central figure for illustration.

Study population, see Fig. 2.

**Fig. 2:**
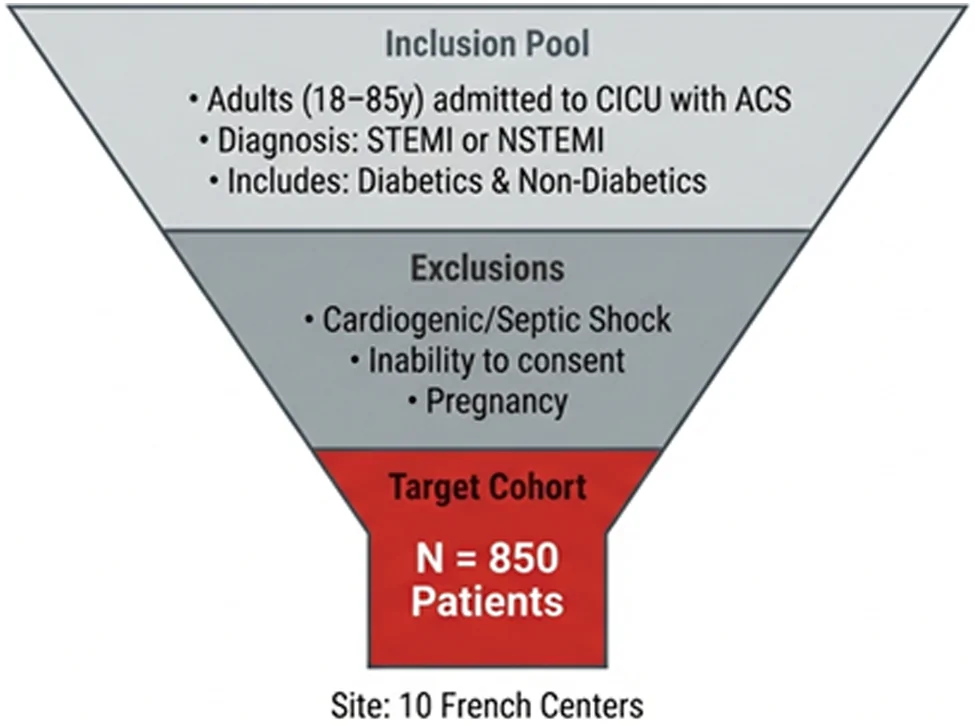
Target population and recruitment logic.

Inclusion criteria:

• Adults aged 18 to 85 years presenting with an acute coronary syndrome (ACS) managed in a cardiac intensive care unit (CICU).
  The diagnosis of ACS will be based on chest pain associated with ST-segment elevation on ECG for ST-segment Elevation Myocardial Infarction (STEMI), or ST-segment changes and/or a positive troponin cycle (values according to the center's protocol) for Non-ST-segment Elevation Myocardial Infarction (NSTEMI).

*Note: Patients with diabetes already using Freestyle Libre (FSL) may be included.*

Exclusion criteria:

• Patients in cardiogenic or septic shock
• Patients who initially presented with ACS managed in a non-investigator center
• Failure to obtain written informed consent signed by both the participant and the investigator upon CICU admission
• Individuals participating in another study with an ongoing exclusion period
• Patients enrolled in a study that could impact post-ACS prognosis
• Individuals deprived of civil rights, under guardianship or trusteeship
• Individuals deprived of liberty (by judicial or administrative decision)
• Individuals whose physical and/or mental health is seriously impaired and who, in the investigator's opinion, may not be able to meet the requirements of the study
• Pregnant or breastfeeding women
• Individuals not affiliated with or not benefiting from a social security system

### 2.3 Procedures

Upon admission, a Freestyle Libre Pro sensor is placed and records interstitial glucose continuously until discharge. Data are downloaded retrospectively. Parameters analyzed include:

• Mean glucose
• Time in range (TIR) (70–180 mg/dL)
• Time above range (TAR) (>180 mg/dL)
• Time below range (TBR) (<70 mg/dL)
• Coefficient of variation (CV)
• Mean Amplitude of Glycemic Excursions (MAGE)
• Glucose management indicator (GMI)

To address the possibility that CGM-derived glycemic variability primarily reflects the severity of the acute coronary syndrome rather than underlying metabolic risk, we will include the type of index event (STEMI, NSTEMI, or other acute cardiovascular diagnosis), the occurrence of cardiogenic shock after enrolment, and routinely available surrogates of infarct size (peak cardiac biomarkers and left ventricular ejection fraction where available) in multivariable models the association between CGM metrics and clinical outcomes.

CGM-derived metrics will be calculated over the entire duration of the in-hospital recording. In our institution, the typical length of stay for uncomplicated ACS ranges from 48 to 72 h, with longer hospitalizations mainly occurring in patients with large infarcts or more severe clinical presentations. In addition to whole-hospitalization analyses, prespecified sensitivity analyses will be performed focusing on the early post-reperfusion period (first 48–72 h after primary PCI or index admission, as appropriate) to specifically capture acute metabolic stress.

Clinical and biological data collected: demographics, Global Registry of Acute Coronary Events (GRACE) and Killip scores, troponin peak, NT-proBNP, echocardiography (Left Ventricular Ejection Fraction [LVEF], strain), coronary anatomy.

### 2.4 Study endpoints and follow-up

The primary endpoint will be the occurrence within 12 months after hospital discharge of a composite endpoint including cardiovascular clinical events: recurrent myocardial infarction (MI), cardiovascular death, stroke, arrhythmias, and/or heart failure requiring hospitalization. The occurrence of these events will be assessed during routine cardiology visits at 1–2 months post-ACS, then at 6 months, and at 12 months.

• Recurrent MI will be defined as the onset of new chest pain with significant troponin elevation and/or ECG changes (as per initial definition).
• Cardiovascular death: The “cardiovascular” nature will be adjudicated by the scientific committee based on collected details. Deaths will be classified as “cardiovascular” or “non-cardiovascular.”
• Stroke will be defined as the occurrence of a neurological deficit lasting more than 24 h, ideally confirmed by brain imaging.
• Arrhythmias will be classified as supraventricular (atrial fibrillation, atrial flutter, other) or ventricular (non-sustained ventricular tachycardia, sustained ventricular tachycardia >30 s, ventricular fibrillation, or other).
• A heart failure hospitalization will be defined by admission with new or worsening symptoms and signs consistent with heart failure (e.g., dyspnea, pulmonary congestion on examination or imaging, peripheral edema), accompanied by objective evidence of cardiac decompensation when available (such as radiographic or echocardiographic findings) and requiring initiation or intensification of heart failure-directed therapy (e.g., intravenous diuretics, vasodilators, inotropes, or escalation of guideline-directed medical therapy). NT-proBNP measurements will be considered supportive but will not be used as a stand-alone criterion to define heart failure events.

The secondary endpoints will be:

• Incidence of diabetes at 1–2 months in patients without diabetes admitted for ACS: defined by fasting plasma glucose ≥126 mg/dL (≥1.26 g/L) and HbA1c ≥6.5%.
• Incidence of diabetes at 12 months in patients without diabetes admitted for ACS: defined by fasting plasma glucose ≥126 mg/dL (≥1.26 g/L) and HbA1c ≥6.5%.

Other data will be collected:

• Clinical data: age, sex, Body Mass Index (BMI).
• Medical history: type 1 or type 2 diabetes, prior ACS, hypertension, hypercholesterolemia, smoking.
• Medications at admission and during hospitalization: cardiovascular drugs, antidiabetic therapy (oral agents, GLP-1 receptor agonists, rapid- or long-acting insulin via subcutaneous injections or IV insulin pump).
• Date of admission to the cardiac intensive care unit (CICU).
• Date of Freestyle Libre Pro sensor placement.
• Continuous Glucose Monitoring (CGM) data: mean interstitial glucose, time in range, time above range, time below range, glycemic coefficient of variation (see details in Section 2.1).
• Biological data: admission plasma glucose, HbA1c.
• Baseline atrial fibrillation (AF) status will be recorded for all participants and categorized as no history of AF, known paroxysmal AF, persistent AF, or permanent AF. New-onset AF episodes occurring during the index hospitalization will be systematically captured. Information on oral anticoagulant therapy (drug class and dose) will be collected at admission and at discharge. AF status and anticoagulation use will be reported descriptively and included as covariates in multivariable models assessing the association between CGM-derived metrics and clinical outcomes, and exploratory analyses will compare patients with pre-existing AF to those with new-onset AF.
• ACS severity:
  (i) *Clinical*: GRACE score (online calculator: http://medicalcul.free.fr/grace.html), Killip classification (classes I–IV).
  (ii) *Biological*: peak plasma troponin (according to center practice, typically between 12 and 48 h), CK if performed, NT-proBNP (first value during hospitalization), serum creatinine (first value during hospitalization).
  (iii) *Imaging*: description of coronary lesions (Left Anterior Descending coronary artery [LAD], Left Circumflex coronary artery [LCx], Right Coronary Artery [RCA]), Thrombolysis In Myocardial Infarction (TIMI) flow classification for each artery.
  (iv) *Echocardiographic parameters*: left ventricular ejection fraction (continuous %), global strain (%).
• Endovascular treatment performed (yes/no), coronary artery(ies) treated.
• Planned coronary bypass surgery (yes/no).
• Discharge medications.
• Date of hospital discharge.
• Date of Freestyle Libre Pro sensor removal.
• During follow-up, occurrence of acute metabolic complications related to diabetes will be assessed: severe hypoglycemia, hypoglycemia requiring hospitalization, hyperglycemia requiring hospitalization. Severe hypoglycemia is defined as requiring assistance from another person to ingest carbohydrates or administer glucagon or intravenous glucose, with or without severe neurological symptoms.

### 2.5 Sample size and statistical plan

#### 2.5.1 Sample size calculation

The sample size calculation is based on the precision of the discriminatory performance of the final model in the development sample, estimated by the Area Under the ROC Curve (AUC).

We expect a cumulative event rate at one year of approximately 5.0%, as observed in the COLCOT study [11]. Assuming a minimum expected AUC of 0.75 and a conservative standard deviation ratio of 1, 800 patients are required to achieve 80% power to demonstrate that the observed AUC (estimated at 0.87) is significantly different from the minimum value, with an alpha risk of 5% (sample size calculated using nQuery/One Sample ROC Curve). To account for an estimated loss to follow-up of 5–6%, 850 participants will be recruited.

### 2.6 Statistical analysis

A detailed statistical analysis plan will be finalized before the end of patient enrolment. The selected approaches are outlined below. Baseline socio-demographic, clinical, and biological characteristics will first be described using means (with standard deviation) or medians (with interquartile ranges) for continuous variables.

The logistic regression model predicting the occurrence of the primary endpoint will be built in several steps using a development sample comprising approximately two-thirds of the cohort (*N* = 567), corresponding to entire centers selected by chronological order of inclusion start (to reduce similarity between development and validation samples).

Among the eight parameters measured by the Freestyle Libre Pro, only one from highly correlated pairs will be retained (e.g., mean interstitial glucose vs GMI, TBR vs hypoglycemic events). Additionally, all variables known to be associated with Major Adverse Cardiac Events (MACE) after ACS (age, sex, admission plasma glucose, admission HbA1c, GRACE score, Killip class, TIMI score, peak troponin, first NT-proBNP value, left ventricular ejection fraction, left main disease) will be considered.

1. **Variable selection in the model**: All candidate variables will be entered simultaneously into a penalized logistic regression using the least absolute shrinkage and selection operator (LASSO), which performs variable selection and coefficient shrinkage in a single step and limits overfitting. The penalty parameter will be tuned by cross-validation within the development sample.
2. **Modeling continuous variables**: Continuous variables will not be dichotomized and will be assumed to have non-normal distributions. They will be transformed using fractional polynomials, logarithmic, power, or square root transformations. If no satisfactory transformation is found, restricted cubic splines will be applied.
3. **Calibration and discrimination**: Model performance will be assessed by calibration (calibration intercept, calibration slope and flexible calibration curve). Discrimination will be evaluated by AUC and its confidence interval, with a minimum expected value >0.75.

### 2.7 Model validation

Given the expected low event rate, the model will be developed on the entire sample (*N* = 850) and validated using bootstrap resampling (internal validation). Validation will be performed on the validation sample (approximately 300 patients) from multiple centers, using calibration and discrimination metrics as described above. The observed AUC in the validation sample will be compared to that in the development sample.

Validation will also be conducted in subgroups (patients with known diabetes before ACS, age ≥ 75 years) as secondary objectives.

Finally, the same development and validation approach will be applied to the secondary objective of predicting incident diabetes at one year. The full penalized procedure will be repeated within each resample.

Special attention will be paid to data quality and endpoint ascertainment. The primary endpoint will be assessed during follow-up visits, particularly at 12 months. If a visit is missed (after reminders), telephone contact with the patient, treating physician, or cardiologist will be made to determine the endpoint status.

### 2.8 Safety analysis

Safety analysis will include participants who had the Freestyle Libre Pro sensor® placed. The incidence of adverse events will be presented according to causality, severity, and intensity. The incidence of adverse events leading to sensor removal will be described, as well as events related to sensor removal.

Adverse events will be categorized according to the hierarchical levels of the Medical Dictionary for Regulatory Activities (MedDRA) classification (latest version at database lock).

### 2.9 Status

The first patient was randomized on November 3, 2023 and enrollment is presently still ongoing.

## 3 Discussion

FREESCA builds upon prior work evaluating CGM in the setting of ACS but differs by its multicenter design, standardized CGM deployment during the acute hospitalization and comprehensive assessment of glycemic metrics. Earlier studies established the feasibility of CGM in ACS and demonstrated its superiority over intermittent capillary testing for detecting transient hyperglycemia, particularly in non-diabetic patients. [12]

Subsequent investigations suggested that CGM-derived indices reflect clinically relevant dysglycemia during hospitalization [13]. More importantly, prognostic studies demonstrated that glycemic variability assessed by CGM is independently associated with adverse cardiovascular outcomes after ACS. Takahashi et al. reported that elevated MAGE was a strong predictor of major adverse cardiovascular and cerebrovascular events over long-term follow-up [14]. Similar associations between glycemic variability and disease progression or outcomes after ACS were reported in other CGM-based analyses. [15]

More recently, contemporary multicenter data have highlighted distinct CGM-derived glucose trajectories after ACS and persistent dysglycemia in both diabetic and non-diabetic patients, reinforcing the potential clinical relevance of CGM beyond the acute phase [16]. Together, these studies support the concept that not only hyperglycemia but also glycemic variability and hypoglycemia may contribute to adverse outcomes through mechanisms involving oxidative stress, endothelial dysfunction and autonomic imbalance.

FREESCA is not positioned as the first study to assess CGM in ACS, but rather as a large, prospective, multicenter effort to harmonize CGM-derived risk markers during hospitalization in a real-world, guideline-based ACS population. Its design allows evaluation of multiple CGM metrics, including clinically relevant hypoglycemia, which has been insufficiently explored in prior ACS-CGM studies.

An important caveat is that patients with larger infarcts, heart failure, or hemodynamic instability are likely to exhibit marked glycemic fluctuations during hospitalization, independently of their baseline metabolic status. In this context, CGM indices might act as integrative markers of ACS severity rather than pure indicators of dysglycemia. Our analytical strategy therefore explicitly adjusts for several dimensions of clinical severity—including infarct type and size, left ventricular function, presence of cardiogenic shock—in order to disentangle, as far as possible, the contribution of metabolic derangement from that of the underlying cardiovascular insult. These additional analyses should help clarify whether CGM-derived variability provides incremental prognostic information beyond conventional measures of ACS severity.

Heart failure will be included in the primary composite endpoint only when it results in hospitalization and meets predefined clinical criteria. A heart failure hospitalization will be adjudicated when the patient is admitted with new or worsening symptoms and signs consistent with heart failure (e.g., dyspnea, pulmonary congestion on examination or imaging, peripheral edema), accompanied by objective evidence of cardiac decompensation when available (such as radiographic or echocardiographic findings) and requiring initiation or intensification of heart failure-directed therapy (e.g., intravenous diuretics, vasodilators, inotropes, or escalation of guideline-directed medical therapy). NT-proBNP measurements will be considered supportive but will not be used as a stand-alone criterion to define heart failure events, in order to avoid misclassification related to nonspecific biomarker elevations in the acute coronary syndrome setting.

A potential limitation of our approach is that CGM metrics are calculated over the entire hospitalization, which may vary in duration according to clinical course and organizational factors. Although the length of stay is generally short (approximately 48–72 h for most ACS patients), later phases of care may still be influenced by changes in treatment and clinical stability rather than by the initial ischemic insult alone. To address this, we will perform complementary analyses restricted to the early 48–72-h period after reperfusion or admission, allowing us to compare the prognostic value of acute-phase versus whole-hospitalization glycemic variability and to better disentangle acute metabolic stress from subsequent management effects.

This study was conceived primarily as a prognostic investigation, without a predefined biobanking strategy or systematic collection of mechanistic biomarkers beyond routine clinical laboratory tests. As a result, potential pathways linking glycemic variability to adverse outcomes—such as oxidative stress, endothelial dysfunction, inflammation, or autonomic dysregulation—cannot be formally tested within the current protocol. Exploratory ancillary analyses based on routinely available biomarkers will be considered, but dedicated mechanistic studies will be required to confirm and refine these hypotheses.

As an observational study, causal inferences cannot be established, and glucose-lowering strategies are not standardized. CGM monitoring is restricted to the hospitalization period, precluding long-term glycemic assessment. Nevertheless, the prospective design, standardized CGM protocol and multicenter recruitment represent major strengths and position FREESCA to provide robust data to inform future interventional trials targeting glycemic management in ACS.

### 3.1 Perspectives

Future research will explore predictive algorithms integrating CGM and clinical data, and randomized trials assessing interventions to reduce glycemic variability.

## Funding and responsibilities

The study is financed by fundings from the General Management Healthcare Provision (DGOS), PHRCI-2021-051. Design of the study was supported by AS, FR; manuscript was written and corrected by ST, FR and AS.

## CRediT authorship contribution statement

**Sarah Tournayre:** Writing – original draft, Project administration, Methodology, Investigation, Formal analysis, Data curation, Conceptualization. **Edouard Gerbaud:** Investigation. **Pierre Poustis:** Investigation. **Bogdan Catargi:** Investigation. **Christel Voinot:** Investigation. **Meyer Elbaz:** Investigation. **Pierre Gourdy:** Investigation. **Benoit Lattuca:** Investigation. **Véronique Taillard:** Investigation. **Patrick Henry:** Investigation. **Jean-François Gauthier:** Investigation. **Frédéric Georger:** Investigation. **Jean-Michel Andrieu:** Investigation. **Etienne Puymirat:** Investigation. **Fabien Huet:** Investigation. **Gwenaelle Arnault:** Investigation. **Nicolas Nagot:** Writing – original draft, Visualization, Validation, Supervision, Resources, Project administration, Methodology, Investigation, Funding acquisition, Data curation, Conceptualization. **François Roubille:** Writing – review & editing, Writing – original draft, Investigation, Funding acquisition, Formal analysis, Data curation, Conceptualization. **Ariane Sultan:** Writing – original draft, Visualization, Validation, Supervision, Software, Resources, Project administration, Methodology, Investigation, Funding acquisition, Formal analysis, Data curation, Conceptualization.

## Ethics

The study was approved by an ethics committee, and all participants provided written informed consent. Conducted in accordance with the Declaration of Helsinki and General Data Protection Regulation (GDPR).

## Declaration of competing interest

The authors declare no conflict of interest.
